# Vitamins D and K as Factors Associated with Osteopathy in Chronic Pancreatitis: A Prospective Multicentre Study (P-BONE Study)

**DOI:** 10.1038/s41424-018-0066-8

**Published:** 2018-10-15

**Authors:** Serena Stigliano, Alexander Waldthaler, Emma Martinez-Moneo, Luana Lionetto, Stuart Robinson, Marko Malvik, Aleksandra Hedstrom, Aleksandra Kaczka, Marius Scholdei, Stephan Haas, Maurizio Simmaco, Gianfranco Delle Fave, Matthias Lohr, Peter Simon, Gabriele Capurso

**Affiliations:** 1 Digestive & Liver Disease Unit, Sant’Andrea Hospital, “La Sapienza”, Rome, Italy; 2 Department for Internal Medicine I, University Hospital Regensburg, Regensburg, Germany; 3 Gastroenterology Department, Cruces University Hospital, Baracaldo, Vizcaya Spain; 4 Mass Spectrometry Laboratory, Sant’Andrea University Hospital, Rome, Italy; 5 Department of HPB Surgery, Newcastle upon Tyne, UK; 6 Department of Endocrinology and Gastroenterology, Tartu University Hospital, University of Tartu, Tartu, Estonia; 7 Gastroenterology and Hepatology, Gastrocentrum, Karolinska University Hospital, Stockholm, Sweden; 8 Department of Digestive Tract Diseases, University Hospital, Lodz, Poland; 9 Department NESMOS, analytical Laboratory Unit, Sant’Andrea Hospital, “La Sapienza”, Rome, Italy; 10 Department of Medicine A, Ernst-Moritz-Arndt-University Greifswald, Greifswald, Germany; 11 Biliary Endoscopy and Endosonography Division, Pancreas Translational & Clinical Research Center, San Raffaele Scientific Institute IRCCS, Milan, Italy

## Abstract

**Background:**

Osteopathy is common in patients with chronic pancreatitis (CP), but previous studies carry several limitations. Vitamin K is essential for bone metabolism, but its role in this setting has never been investigated. Our aim is to assess the prevalence of osteoporosis and osteopenia in CP patients, and to investigate the association between osteopathy and CP features and nutritional parameters, especially vitamin D and K levels.

**Methods:**

Multicentre cross-sectional study on CP patients diagnosed according to M-ANNHEIM criteria. Bone density was evaluated by dual-energy X-ray absorptiometry and pancreatic function by faecal elastase. Nutritional evaluation included vitamin D and vitamin K. Differences between patients with or without osteopathy were evaluated. The association between investigated variables and bone density were analysed with logistic regression analysis.

**Results:**

In total, 211 CP patients were enrolled at eight Centres (67% men; mean age 60). In total, 18% had advanced-marked CP, 56% suffered from pancreatic exocrine insufficiency and disease aetiology was alcoholic in 43%. Vitamin D and K were deficient in 56% and 32%, respectively. Osteopenia was diagnosed in 42% and osteoporosis in 22%. In the multivariate analysis, female sex (OR 2.78), age (OR 1.07 per year) and higher BMI (OR 0.84) were associated with the presence of osteoporosis. In male patients, the only factor associated with osteoporosis was vitamin K deficiency (OR 4.23).

**Conclusion:**

The present data confirm a high rate of osteopathy in CP patients and highlight the relevance of vitamin K deficiency as only factor associated with osteoporosis in male patients for the first time.

## Background

Chronic pancreatitis (CP) is a fibro-inflammatory syndrome of the pancreas characterised by irreversible morphological changes that typically cause abdominal pain and can eventually result in permanent loss of the pancreatic function^1,2^. The clinical presentation depends on disease stage, as in early phases episodes of abdominal pain are more frequent while, as the disease progresses, signs of pancreatic exocrine and endocrine insufficiency develop^1,3^. Malnutrition due to exocrine pancreatic insufficiency, as well as alcohol abuse, chronic inflammation, and tobacco use can contribute to systemic complications in CP patients, including bone alterations^4^.

Osteoporosis is a preventable disease characterised by low bone mass and micro-architectural deterioration of bone tissue with a consequent increase in bone fragility^5^. It negatively affects quality of life and is associated with an increased susceptibility to fractures, which are accompanied by considerable morbidity and mortality. A number of pathological conditions, such as primary hyperparathyroidism, hyperthyroidism, chronic kidney failure, chronic liver disease, different digestive disorders and the use of drugs such as glucocorticoids are associated to accelerated bone loss and development of osteopenia and osteoporosis^5^.

In particular, low bone mineral density (BMD) is a common finding in chronic intestinal disorders associated with diarrhoea and malabsorption, such as inflammatory bowel diseases and coeliac disease^6,7^.

Changes in BMD are, however, also frequent in CP patients^4^. The prevalence of osteoporosis in CP patients has been reported to range from 5 to 39%^8–11^, and a meta-analysis estimated that about 25 and 40% of CP patients are affected by osteoporosis and osteopenia, respectively^12^. Indeed, the recent European guidelines for the diagnosis and treatment of CP recommend regular bone density assessment by dual-energy X-ray absorptiometry (DXA), and measurement of serum vitamin D as part of CP patients' primary care^1^.

BMD alterations of in CP patients might be caused by maldigestion and malabsorption of micronutrients and of fat-soluble vitamins like vitamin D and K, by the chronic inflammatory status and by excessive alcohol intake and smoking^13–16^.

However, the pathogenesis of bone density alterations in CP patients is not well established as previous studies carry several limitations. First, most studies had a very small sample size (the largest including 100 patients); moreover, different and heterogeneous nutritional parameters were evaluated and pancreatic exocrine insufficiency (PEI) or severity of the disease were not consistently defined^8,10,11,17–24^. No clear correlation between pancreatic function, disease severity, nutritional parameters and lifestyle factors were found^8,10,11,17–24^. Finally, none of the previous studies investigated vitamin K levels.

Vitamin K is a fat-soluble vitamin and may therefore be malabsorbed in CP patients. However, only few studies evaluated vitamin K levels in CP patients with findings of vitamin K deficiency in more than 60% of cases^25–27^. In particular, there have been no previous studies examining the association between vitamin K levels and bone metabolism in CP patients.

Vitamin K is essential for bone metabolism since it is a cofactor for the enzyme gamma glutamyl carboxylaxe, which converts osteocalcin (OC) to its carboxylated form. Fully carboxylated OC has a high affinity for calcium and hydroxyapatite and binds calcium to the hydroxyapatite crystal in the extracellular bone matrix^28,29^. Several studies have shown a key role of vitamin K deficiency in causing low BMD in patients with other pathological conditions, including inflammatory bowel disease^30,31^, but there are no studies investigating this association in CP patients. Hence, the aims of this study were to asses in a large and homogeneously defined cohort of patients with CP: (a) the prevalence of osteoporosis and osteopenia; (b) the association between bone mineral density and CP features, and nutritional parameters, especially vitamin D and K levels.

## Patients and Methods

### Study design and population

This is a multicentre cross-sectional study conducted in Estonia, Germany, Italy, Poland, Spain, Sweden and the United Kingdom, from April 2015 to October 2016, as part of the “Pancreas 2000” educational program”. Cases of CP seen at the participating Centres during the study period were eligible for enrolment. Enrolled patients gave written informed consent. Local ethic committee approval was obtained at each Centre.

Inclusion criteria were age > 18 years and a diagnosis of probable or definite CP according to the M-ANNHEIM criteria^32^. Patients with a diagnosis of pancreatic cancer, liver cirrhosis, chronic renal failure, primary hyperparathyroidism, small bowel, gastric or pancreatic surgery, celiac disease or inflammatory bowel disease were excluded. Chronic corticosteroids use ( ≥ 7.5 mg/dl for > 3 months) was also considered an exclusion criterion. Patients were not enrolled during acute episodes of exacerbation of symptoms of CP leading to inpatient hospital stay. The severity of CP was defined according to the M-ANNHEIM scoring system^32^. Patients were questioned on epidemiological data, environmental risk factors and lifestyle, history and course of disease, occurrence of menopause, history of previous low fragility/trauma fractures, defined as those occurring at the spine, hip and distal radius, and not associated with traumatic events^9^ and use of pancreatic enzyme replacement therapy (PERT) by a medical doctor in their native language. In keeping with ADA guidelines, patients were diagnosed with diabetes mellitus if they had either: (a) a HbA1c value ≥ 6.5% or a fasting blood glucose ≥ 126 mg/dL, or a 2 h post-OGTT glucose level ≥ 200 mg/dl^33^. Diabetics were asked about the date of diabetes diagnosis. A consumption of at least 12.5 g (1 unit) of alcohol/month was needed to be considered a drinker. One glass of wine, 1 pint or can of beer, one shot of hard liquor were each considered approximatively equal to 1 unit. For smokers, a consumption of at least 100 cigarettes or > 6 months of smoking was needed to be considered a smoker. The total amount of smoking was evaluated as pack-years, defined as the product of packs smoked per day and the total years of smoking. Body mass index (BMI) was calculated as usual adult weight/height^2^ (kg/m^2^)^34^.

### Bone mineral density and biochemical evaluation

Standardized osteodensitometry by DXA and biochemical tests were performed at each participating Centre within 6 months from the enrolment visit. DXA evaluated the BMD in the lumbar spine (L1–L4) and in the left femoral neck (except in patients with a left hip prosthesis or former fracture). Bone mass measurements results are reported as T-score, reflecting the number of standard deviations above or below the mean for a young adult population. Osteopenia was defined as a T-score < −1 and osteoporosis was defined as a T-score < −2.5^35^.

In the reported analysis for the outcome “osteoporosis” in the case of patients having both osteopenia and osteoporosis at different body sites, we considered the worst condition to classify the patient.

PEI was estimated with faecal elastase-1 using a monoclonal enzyme-linked immunosorbant assay (ScheBo Pancreatic elastase-1 stool test, Biotech AG, Giessen, Germany) at each Centre. Results were classified as normal pancreatic function (> 200 mcg/g), mild PEI (100 to 200 mcg/g) and severe PEI (< 100 mcg/g)^36^. Vitamin D (25-OH cholecalciferol), total and ionized calcium, magnesium, parathyroid hormone (PTH) and c-reactive protein (CRP) were measured locally at each Centre. Vitamin D deficiency was diagnosed when levels were < 20 ng/ml.

### Vitamin K determination

Determination of vitamin K levels (K1, Phylloquinone) in the serum samples was centralized in the laboratories of S. Andrea Hospital, University “Sapienza” (Rome, Italy). Blood specimens for vitamin K determination were taken from patients fasting from at least 12 h, and immediately centrifuged at 4000 revolutions per minute to obtain sera and frozen at –80 °C until analysis. Details regarding ultra high performance liquid chromatography (HPLC) methods of vitamin K measurement are provided as Supplementary material. The lower limit to define for vitamin K deficiency was set at 0.2 ng/ml considering the values between the 5th and the 95th percentile of the distribution of a group of 20 healthy controls previously employed to set the method. Notably, this cut-off of ng/ml resulted in line with those reported by others^37,38^.

### Statistical analysis

The prevalence of osteoporosis or osteopenia was calculated dividing the patients with such pathological condition by the total number of participants. Differences in terms of demographics, clinical characteristics, risk factors and nutritional factors between patients with or without osteoporosis or osteopenia were evaluated trough Fisher exact test for categorical variables and *t* test for continuous variables.

The influence of nutritional and clinical parameters on BMD was evaluated throughout a logistic regression analysis with enter method, and odds ratio (OR) and their 95% confidence intervals (CI). The outcome variables were either osteopenia or osteoporosis, and the explored explanatory variables were: age (per increasing year); female sex; BMI (per increasing unit); PTH (per increasing unit); vitamin D deficiency; vitamin K deficiency; CRP (per unit); faecal elastase (per increasing unit); diabetes; active smoking; alcoholic aetiology; disease duration (years), use of PERT.

All analyses were corrected for the Centre of enrolment. Centre of enrolment was, indeed, considered as a categorical covariate in all logistic regression analyses, assigning each individual patient to a nominal category on the basis of the Centre where the patient was enrolled.

As osteopathy has peculiar mechanisms that are related with hormonal factors, a subgroup analysis by either female or male sex was planned. The possible interaction between vitamin D and vitamin K deficiencies and the diagnosis of osteoporosis was explored. The possible correlation between continuous variables was assessed throughout a Pearson correlation test. All reported *P*-values are two-sided. *P*-values < 0.05 were considered statistically significant. Statistical analysis was performed using MedCalc version 13 (MedCalc Software, Belgium).

## Results

### Demographics and clinical features

In total, 211 patients with CP were enrolled (see Table 1): 58 (27.48%) in Italy, 57 (27%) in Germany, 29 (13.74%) in Sweden, 28 (13.27%) in Spain, 15 (7.10%) in Estonia, 13 (6.16%) in Poland and 11 (5.21%) in England; 67% were male with a mean age of 60 years. The mean disease length from diagnosis to enrolment was 75.21 months (95% CI 63.08–87.33); 60% of the patients had a history of alcohol drinking and 28% of them were active drinkers at enrolment. The aetiology was considered alcoholic in 43.6% of cases. Overall, 145/211 (69%) of patients had a history of smoking and 70% of them were heavy smokers as defined by > 20 pack years. Diabetes Mellitus was present in 77/211 (37%) of cases. Only 55 of these diabetic patients were able to report the exact date of diabetes diagnosis, permitting to define 37/77 (48%) as with type II diabetes and 18/77 (23%) as with diabetes of the exocrine pancreas, while this information was missing for the remaining 22/77 (29%).The mean BMI at the time of enrolment was 24 ± 4. Of the 69 female patients, 61/211 (28.9%) were in menopause.

**Table 1:** General features of the enrolled cohort of CP patients

Patient’ s features	Number (%)
*Sex*	
Male	142/211 (67.29%)
Female pre-menopause	8/211 (3.79%)
Female post-menopause	61/211 (28.90%)
Body mass index (meaan)	24 ± 4
Diabetes	77/211 (37%)
Ever alcohol drinkers	127/211 (60%)
Ever smokers	145/211 (69%)
*Aetiology*	
Alcoholic	92/211 (43.60%)
Idiopathic	40/211 (18.95%)
Hereditary	9/211 (4.26%)
Obstructive	12/211 (5.68%)
Other	58/211 (27.48%)
*Disease Severity*	
Minor	74/211 (35%)
Increased	99/211 (47%)
Advanced	32/211 (15%)
Marked	6/211 (3%)
*Pancreatic Exocrine Function*	
Normal	78/179 (43.57%)
Faecal elastase < 200 mcg/g > 100 mcg/g	29/179 (16.20%)
Faecal elastase < 100 mcg/g	72/179 (40.22%)
Pancreatic enzyme replacement therapy	116/211 (54.97%)
*Bone mineral density*	
Normal	76/211 (36%)
Osteopenia	89/211 (42.18%)
Osteoporosis	46/211 (21.80%)
*Vitamins dosage*	
Vitamin D (mean value; ng/ml)	20.2 ± 12
Vitamin D deficiency ( < 20 ng/ml)	119/211 (56.39%)
Vitamin K (mean value; ng/ml)	0.64 ± 0.9
Vitamin K deficiency ( < 0.2 ng/ml)	56/178 (31.46%)

Data are presented as rate or as mean ( ± SD)

Faecal elastase levels were measured within 6 months from enrolment in 179/211 patients (85% of the study population). in total,101 patients (56.42%) had PEI. Faecal elastase was between 200 mcg/g and 100 mcg/g in 29/179 (16.20%) and < 100 mcg/g in 72/179 (40.22%). Overall, 116/211 patients (54.97%) were receiving treatment with PERT. According to the M-ANNHEIM classification, 18% of patients were classified as advanced or marked CP.

### Rate of osteopathy

Overall, 135/211 patients had osteopathy (64%). Eighty-nine of the 211 (42.18%) patients had osteopenia at any site as the worst bone damage, while 46/211 (21.80%) had osteoporosis that was located at the lumbar spine in 26% of cases, at the femoral neck in 39% and at both sites in 35%. The rate of osteoporosis was 0% (0/15) in Estonia, 17% (5/29) in Sweden, 18% (2/11) in England, 21% (12/57) in Germany, 21.4% (6/28) in Spain, 23% (3/13) in Poland and 31% (18/58) in Italy.

Previous low-trauma pathological fractures were reported by 13/196 (6.63%) of patients who were able to recall these events. The remaining 16 patients were unsure about these events.

### Vitamin D levels

Vitamin D was quantified within 6 months from enrolment in 204/211 patients (96.68%). The mean value of vitamin D was 20.2 ng/ml, and 115/204 (56.37%) had vitamin D deficiency ( < 20 ng/ml). The rate of vitamin D deficiency was 73% (11/15) in Estonia, 54% (15/28) in Sweden, 90% (9/10) in England, 51% (29/57) in Germany, 61% (17/28) in Spain, 100% (11/11) in Poland and 40% (22/55) in Italy.

### Vitamin K levels

Serum samples for Vitamin K levels analysis were taken within 6 months from enrolment in 178/211 (84.36%) patients. The median Vitamin K concentration was 0.38 ng/ml (range 0.008–7.8). In total, 56 of the 178 CP patients (31.46%) had Vitamin K deficiency (< 0.2 ng/ml). The rate of vitamin K deficiency was 40% (6/15) in Estonia, 12.5% (3/24) in Sweden, 38% (21/55) in Germany, 37% (10/27) in Spain, 55% (6/11) in Poland and 22% (10/46) in Italy.

Of the 56 patients with vitamin K deficiency, 37 (66%) were male and 47% had alcoholic aetiology of the disease. Only 9% of patients with vitamin K deficiency had advanced or marked CP, while 48% of them had PEI.

In addition, a combined deficiency of vitamin K and vitamin D was observed in 33 of 178 patients (18.53%). Patients with combined deficiency of both vitamin D and K did not differ from those without vitamin deficiencies or single vitamin deficiency, but had a higher rate of alcoholic aetiology (60.6% vs. 39.4% *P=0.05*).

### Other biochemical features

Overall, 29/180 patients (16.11%) had magnesium deficiency and 24/193 (12.43%) calcium deficiency, 18.28% of patients (32/175) had PTH levels above the upper normal range and 70/115 (60.86%) had CRP levels above the upper normal range.

### Association between patients’ characteristics, vitamin D and K levels, and osteopathy

There were no differences between patients with or without osteopenia in terms of sex, age, length and severity of disease, presence of PEI and vitamin D or K insufficiency rate.

The mean BMI (24 ± 3 vs. 25 ± 3; *P* = 0.05) and the rate of diabetes (31% vs. 46%; *P* = 0.05) were lower in CP patients with osteopenia (Table 2).

**Table 2:** Differences among CP patients with normal bone mineral density, osteopenia or osteoporosis

	Normal (*n*=76)	Osteopenia (*n*=89)	Normal vs. Osteopenia *P*-value	Osteoporosis (*n*=46)	Normal vs. Osteoporosis *P*-value
Age (mean)	55 ± 12	59 ± 13	0.06	67 ± 9	0.0001
Female sex	17/76 (22%)	26/89 (29.2%)	0.37	26/46 (56.6%)	0.0001
Menopause	10 / 14 (71.4%)	22/26 (84.6%)	0.41	25/25 (100%)	0.012
Body mass index (mean)	25 ± 4	24 ± 3	0.05	22 ± 3	0.001
Pathological fractures	2/68 (2.9%)	8/86 (9.3%)	0.18	3/42 (7.1%)	0.36
Length of disease (mean)	81 ± 97	76 ± 79	0.76	98 ± 99	0.38
Advanced-marked disease	14/67 (20.8%)	17/85 (20%)	1	4 /41(9.7%)	0.18
Diabetes	35/76 (46%)	27/87 (31%)	0.05	15/45 (33.3%)	0.18
FE < 200 mcg/g > 100 mcg/g	37/63 (58.7%)	44/76 (57.9%)	1	20/40 (50%)	0.42
FE < 100 mcg/g	25/63 (39.6%)	34/76 (44.7%)	0.6	13/40 (32.5%)	0.53
PERT	37/76 (48.6%)	54/89 (60.6%)	0.15	25/45 (55%)	0.57
Active smoking	40/76 (52%)	42/87 (48.2%)	0.63	22/44 (50%)	0.85
Pack years > 20	30/45 (66%)	32/46 (69.5%)	0.82	18/23 (78%)	0.40
Alcoholic aetiology	36/76 (47.3%)	37/87 (42.5%)	0.63	18/45 (40%)	0.45
Vitamin D deficiency ( < 20 ng/ml)	43/74 (58.1%)	50/87 (57.5%)	1	22/43 (51.2%)	0.56
Vitamin D levels ng/ml (mean)	19 ± 12	19 ± 12	0.86	23.1 ± 14	0.13
Vitamin K deficiency ( < 0.2 ng/ml)	20/65 (30.7%)	23/77 (29.8%)	1	13/36 (36.1%)	0.66
Vitamin K levels ng/ml (mean)	0.57 ± 0.58	0.79 ± 1.18	0.17	0.44 ± 0.47	0.26
Mg levels mg/dl (mean)	1.82 ± 0.39	1.77 ± 0.44	0.49	1.84 ± 0.48	0.78
Mg deficiency	9/66 (13.6%)	14/76 (18.4%)	0.49	6/38 (15.7%)	0.77
Ca levels mg/dl (mean)	8.78 ± 1.77	9.2 ± 1.96	0.92	9.28 ± 1.35	0.52
Calcium deficiency	9/69 (13%)	10/83 (12%)	1	5/43 (11.6%)	1
PTH levels pg/ml (mean)	43 ± 16	50 ± 23	0.06	70 ± 62	0.002
PTH above normal	4/60 (6.6%)	15/79 (18.9%)	0.04	13/36 (36%)	0.001
CRP levels mg/dl (mean)	6.4 ± 16	3 ± 6	0.15	2.46 ± 4	0.29
CRP above normal	28/44 (63.6%)	32/52 (61.5%)	1	10/19 (52.6%)	0.57

Data are presented as rate or as mean ( ± SD)

CP patients with osteoporosis were more frequently female (56.6% vs. 22%; *P* = 0.0001) and their mean age was higher (67 ± 9 years vs. 55 ± 12 years; *P* = 0.0001) compared with those without. In addition, all women with osteoporosis were post-menopausal compared with 70% of those without osteoporosis (*P* = 0.01).

The mean BMI was lower in patients with osteoporosis compared with those without (22 ± 3 vs. 25 ± 4; *P *= 0.001). CRP levels were not different between patients with normal bone density, osteopenia or osteoporosis.

Neither vitamin D nor vitamin K levels were statistically different between patients with and without osteoporosis, while the mean value of PTH was significantly higher (70 ± 62 pg/ml vs. 43 ± 16 pg/ml; *P* = 0.002) in patients with osteoporosis.

The rate of pathological fractures was not significantly different in patients with osteoporosis compared with the normal group (7% vs. 2.9%; *P* = 0.36) (Table 2).

A logistic regression analysis was run to investigate the possible association between the analysed variables and the diagnosis of osteopathy (either osteopenia or osteoporosis), osteopenia or osteoporosis. Factors significantly associated with osteopathy in the univariate logistic regression analysis were: female sex (OR 2.17 95% CI 1.14–4.12; *P* = 0.014), a higher age (OR 1.04 per increasing year; 95% CI 1.01–1.06; *P* = 0.0005), a higher BMI (OR 0.89 per unit; 95% CI 0.83–0.96; *P* = 0.003) and a higher PTH (OR 1.01 per unit; 95% CI 1–1.03; *P* = 0.02) and a diagnosis of diabetes (OR 0.54; 95% CI 0.30–0.97; *P* = 0.04). Age (OR 1.04 per increasing year; 95% CI 1.01–1.07; *P* = 0.002), a higher BMI (OR 0.89 per increasing unit; 95% CI 0.81–0.97; *P* = 0.01) and a higher PTH (OR 1.01 per increasing unit; 95% CI 1.01–1.03; *P* = 0.03) were maintained as significant factors in the multivariate analysis.

There were no factors significantly associated with osteopenia at the multivariate logistic regression analysis.

As far as osteoporosis, in the univariate analysis, female sex (OR 3.44 95% CI 1.70–6.96; *P* = 0.0005) and higher age (OR 1.06 per increasing year; 95% CI 1.03–1.10; *P* = 0.0002) were associated with a higher risk, while a higher BMI (OR 0.84 per unit; 95% CI 0.76–0.94; *P* = 0.001) was associated with reduced risk.

The same factors were significantly associated with osteoporosis also in the multivariate analysis. In particular, female sex (OR 2.78 95% CI 1.16–6.68; *P* = 0.021) and higher age (OR 1.07 per increasing year; 95% CI 1.02–1.11; *P* = 0.001) were associated with a higher risk of being diagnosed with osteoporosis while a higher BMI (OR 0.84 per unit; 95% CI 0.74–0.95; *P* = 0.006) was associated with reduced risk (see Table 3).

**Table 3:** Logistic regression analysis for factors associated with osteoporosis in the whole population of 211 patients with CP

Univariate^a^			Multivariate^a^	
	OR (95% CI)	*P*-value	OR (95% CI)	*P*-value
Age (per increasing year)	1.06 (1.03–1.10)	0.0002	1.07 (1.02–1.11)	0.001
Female sex	3.44 (1.70–6.96)	0.0005	2.78 (1.16–6.68)	0.021
BMI (per increasing unit)	0.84 (0.76–0.94)	0.001	0.84 (0.74–0.95)	0.006
PTH (per increasing unit)	1.01 (1.00–1.01)	0.01	1.01 (1.00–1.03)	0.037
Vitamin D deficiency	1.10 (0.53–2.27)	0.20	–	
Vitamin K deficiency	1.45 (0.64–3.29)	0.18	–	
CRP (per unit)	0.98 (0.88–1.08)	0.70	–	
Faecal elastase (per increasing unit)	1.00 (0.99–1.00)	0.90	–	
Diabetes	0.81 (0.40–1.63)	0.66	–	
Active smoking	1.10 (0.54–2.23)	0.77	–	
Advanced-marked disease	0.47 (0.14–1.59)	0.22	–	
Alcholic aetiology	0.82 (0.41–1.60)	0.56	–	
Disease duration (per year)	1.00 (0.99–1.00)	0.18	–	
PERT	1.01 (0.52–1.97)	0.96	–	

*BMI* body mass index, *PTH* parathormone, *CRP* C-reactive protein, *PERT* pancreatic enzyme replacement therapy

^a^Adjusted for centre of enrolment

As female patients in the post-menopausal age are likely to have distinct causes for osteopathy and as most CP patients are male, we repeated the analysis for osteoporosis considering female and male patients separately. In male CP patients, a higher BMI (OR 0.84 per unit 95% CI 0.72–0.97; *P* = 0.02) was associated with a lower risk of being diagnosed with osteoporosis while age was only a borderline significant factor (OR 1.04 per year; 95% CI 0.99–1.09; *P* = 0.06). In addition, vitamin K deficiency was the only variable significantly associated with a higher risk in the multivariate analysis (OR 5.28; 95% CI 1.31–21.4; *P* = 0.01) (Table 4).

**Table 4:** Logistic regression analysis for factors associated with osteoporosis in the 142 male patients with CP

Univariate^a^			Multivariate^a^	
	OR (95% CI)	*P*-value	OR (95% CI)	*P*-value
Age (per increasing year)	1.04 (0.99–1.09)	0.06	–	
BMI (per increasing unit)	0.84 (0.72–0.97)	0.02	0.85 (0.71–1.03)	0.11
PTH (per increasing increasing unit)	1.01 (1.00–1.03)	0.04	1.01 (0.99–1.03)	0.22
Vitamin D deficiency	1.14 (0.38–3.44)	0.81	–	
Vitamin K deficiency	4.25 (1.27–14.2)	0.01	5.28 (1.31–21.40)	0.01
CRP (per increasing unit)	0.84 (0.59–1.21)	0.36	–	
Faecal elastase (per increasing unit)	1.00 (0.99–1.00)	0.29	–	
Diabetes	0.47 (0.15–1.47)	0.19	–	
Active smoking	1.45 (0.48–4.35)	0.50	–	
Advanced-marked disease	0.52 (0.10–2.67)	0.44		
Alcholic aetiology	1.04 (0.37–2.94)	0.93	–	
Disease duration (per year)	1.00 (0.99–1.00)	0.81	–	
PERT	0.82 (0.31–2.11)	0.68	–	

*BMI* body mass index, *PTH* parathormone, *CRP* C-reactive protein, *PERT* pancreatic enzyme replacement therapy

^a^Adjusted for centre of enrolment

To investigate whether there was a an interaction between vitamins D and K deficiencies and osteoporosis, we conducted a further analysis on male CP patients, investigating those with vitamin D or K deficiency only or their combined deficiency separately (see Supplementary Table 1). Of note, there was a higher rate of vitamin K deficiency only in patients with osteoporosis (27.7%) compared with those without osteoporosis (5.9%) with a significant association at the adjusted logistic regression analysis (OR 9.21; 95% CI 1.89–44.79; *P* = 0.005). This was not the case for isolated deficiency of vitamin D or for combined deficiency of vitamins D and K, which were not associated with the diagnosis of osteoporosis.

In female patients, only increasing age was significantly associated with the diagnosis of osteoporosis in the multivariate analysis (OR 1.09 per increasing year; 95% CI 1.03–1.16; *P* = 0.002) (Supplementary Table 2).

**Fig. 1:**
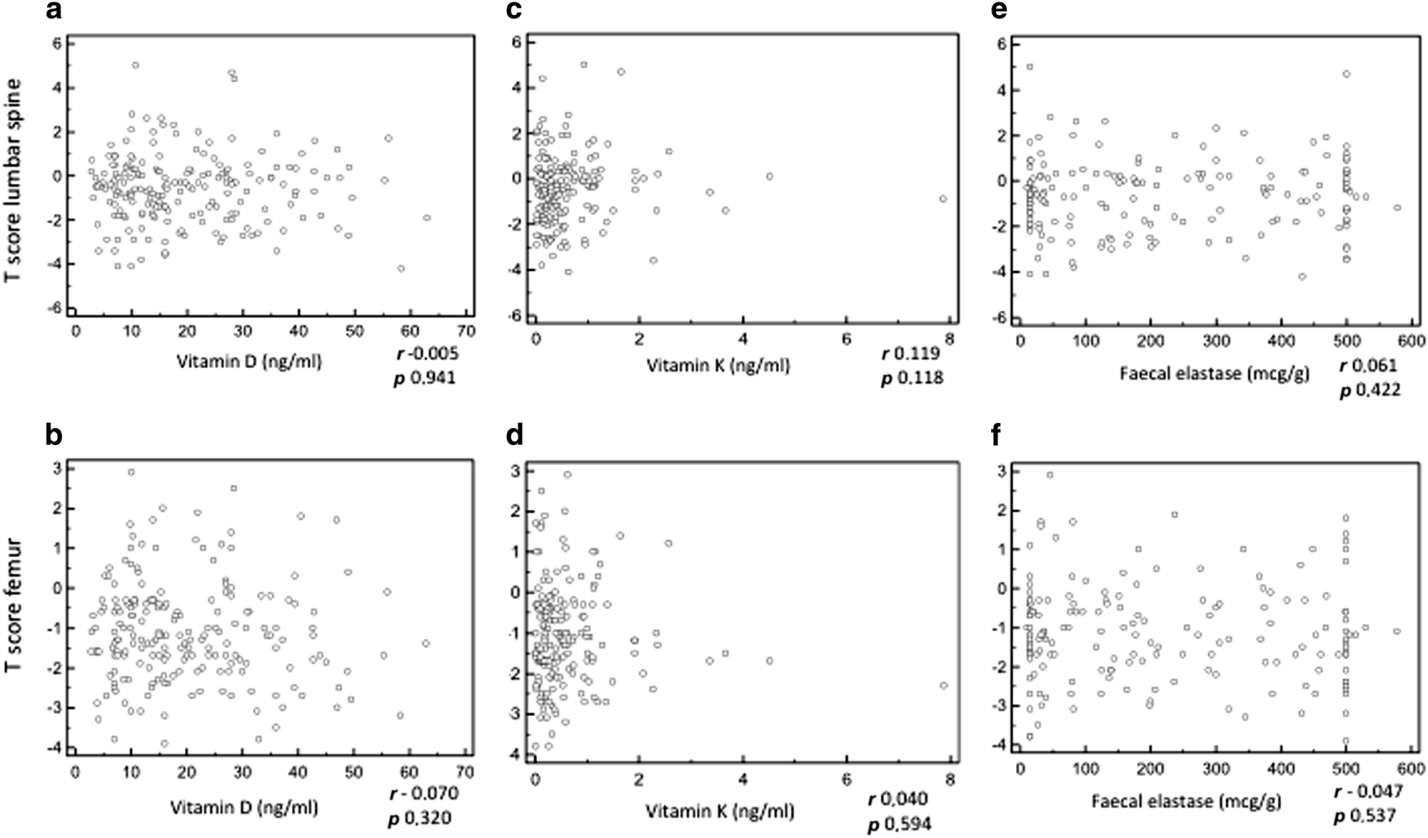
Correlation analysis. Correlation between: vitamin D levels and T-score at the lumbar spine (**a**), vitamin D levels and T-score at the femur neck (**b**), vitamin K levels and T-score at the lumbar spine (**c**), vitamin K levels and T-score at the femur neck (**d**), faecal elastase levels and T-score at the lumbar spine (**e**), faecal elastase levels and T-score at the femur neck (**f**). The Pearson correlation test was not significant in all cases

There was no correlation between vitamin D, vitamin K and faecal elastase levels and T score in femoral neck or lumbar spine (Fig. 1, panels a–f).

## Discussion

Osteoporosis is common in CP patients, but the reported prevalence is heterogeneous, ranging from 5% to 39%^8,10,11,17–24^, with a pooled rate of 23% in a meta-analysis^12^. Nevertheless, previous studies are limited by the small sample size and the lack of a consistent definition of criteria for CP diagnosis and of assessment of severity and PEI. Furthermore, while some studies investigated the possible role of Vitamin D deficiency among factors associated with osteoporosis, none of them investigated vitamin K levels. Both vitamin D and vitamin K are lipid-soluble and are frequently malabsorbed in CP patients, contributing to the development of osteopathy^26^.

This is the largest study investigating the prevalence of osteopathy in CP patients. It was conducted in a prospective multicentre setting with standardized criteria to assess disease severity and pancreatic insufficiency on a final population of 211 CP patients. Furthermore, the potential role of vitamin K deficiency as causative factor for osteopathy in CP was analysed for the first time (Tables 1 and 2). The present findings of a 42% rate of osteopenia and 22% of osteoporosis are consistent with previous studies, suggesting the genuineness of our CP population. These results strengthen recent recommendation of guidelines for active screening for osteopathy in patients with CP^1^. In our population, about half of the patients had vitamin D insufficiency and almost 60% had PEI, but, collectively, these factors were not associated with osteopathy. Indeed, at the multivariate analysis, only female sex, older age and lower BMI were associated with an increased risk of being diagnosed with osteoporosis (Table 3). As all female patients with osteoporosis were post-menopausal, we repeated our analyses considering only male subjects, who represent the majority of our cohort as in any CP population. Interestingly, in male CP patients vitamin K deficiency resulted the only factor associated with a higher risk of being diagnosed with osteoporosis at the multivariate analysis (Table 4).

Vitamin K is a fat-soluble vitamin that plays a key role in normal bone metabolism. Vitamin K deficiency has, indeed, been reported to be a frequent finding in subjects with osteoporosis and pathological fractures^39^. Vitamin K deficiency has also been associated with osteopathy in patients with Crohn’s disease and, more interestingly, in subjects with cystic fibrosis who often suffer from PEI^40^. The median vitamin K concentration in patients with Crohn’s disease^30^ has been reported to be 0.4 ng/ml, a figure that is very close to the median of 0.38 ng/ml observed in our CP cohort.

Our findings of a 32% rate of vitamin K deficiency and of its association with osteoporosis in male CP patients suggest that vitamin K supplementation should be considered alongside vitamin D in CP patients. Vitamin K exists in several isoforms. Phylloquinone (vitamin K1) is the most prevalent form in the human diet, while Menaquinone (vitamin K2) is mainly synthetized by the gut microbiome. Whether this “microbiome-generated” fraction is active in a similar manner to vitamin K1 is uncertain^28^, and, although the action of vitamin K2 seems more specific for bones, all vitamin K isoforms have a role as cofactors in the conversion of vitamin K-dependent proteins, such as prothrombin and osteocalcin, from an inactive to an active form. We therefore chose to measure vitamin K1 levels as they might more reliably be related to dietary intake and absorption, which are hampered in CP patients^28^. As the gut microbiome is altered in patients with CP as well^41^, future studies should also focus on its investigation and on the relation between vitamin K2 levels and bone metabolism.

Furthermore, we investigated the possible correlation between levels of vitamins D and K, faecal elastase and the T-score at femur and spine, without significant results (Fig. 1). The present findings of no correlation between vitamin D levels and osteopathy are not surprising, and result in accordance with the majority of the previous studies on this topic^8,18,19^. Similarly, a relation between faecal elastase levels and BMD has been reported in some previous studies^11,19,22,24^, but not in others^8,17,21^. This might be due to impaired micronutrient absorption being only one of several factors leading to osteopathy in CP patients, and to most cases with PEI receiving PERT. Further prospective studies including only newly diagnosed patients without prior medication might clarify this issue. On the other hand, the present finding of a lower BMI being associated with a lower BMD is in accordance with previous findings^8,23^.

Interestingly, a number of experimental and observational studies have suggested that vitamins D and K might act synergistically on bone health, as vitamin D seems able to enhance vitamin K-dependent proteins^42^. We, therefore, explored whether there was an interaction between vitamins D and K deficiencies and osteoporosis, analysing the rate of osteoporosis in male CP patients with vitamin D or K deficiency only or with combined deficiency separately (see Supplementary Table 1). Of note, there was a higher rate of vitamin K deficiency only in patients with osteoporosis (27.7%) compared with those without osteoporosis (5.9%) with a significant association at the adjusted logistic regression analysis (OR 9.21; 95% CI 1.89–44.79; *P* = 0.005). This was not the case for isolated vitamin D deficiency or for combined deficiency of vitamins D and K. These findings do not support the hypothesis of an interaction between vitamin D and K deficiencies in determining an increased risk of osteoporosis in this setting, but this topic deserves further investigation.

A relevant role for CRP, a marker of acute phase reaction, has been recently reported not only as a prognostic variable associated with CP course^43^, but also as a marker of decreased bone density^4^. In the present study, however, CRP levels were not associated with BMD. This difference might be due to the fact that this previous study evaluated high sensitivity CRP levels, which better reflects low-grade chronic inflammation, rather than CRP as in the present one.

As expected, PTH levels were higher in patients with osteopathy and associated with osteoporosis in the whole investigated population (Tables 2 and 3), but not in the sub-analysis conducted in male patients only (Table 4). Secondary hyperparathyroidism in CP patients is due to decreased absorption of calcium and vitamin D and can, in turn, determine increased bone remodelling contributing to the development of osteoporosis^44^.

The present study has some strengths: (1) the sample size is by far the largest for studies of this kind; (2) data were collected in a homogeneous and standardised way, the diagnosis of CP and its severity being defined according to the M-ANNHEIM classification; (3) exocrine pancreatic insufficiency was investigated through measurement of faecal elastase levels, which is nowadays the most commonly employed test for this aim; (4) different clinical and biochemical variables potentially associated with osteopathy were analysed including a centralized quantification of vitamin K, which was investigated for the first time in this setting.

There are, however, some limitations which are partly intrinsic to the study setting of a multicentre cross-sectional study. Some investigations, such as DXA, and measurement of all biochemical parameters but vitamin K were performed at each local institution, and this might have caused bias due to different methods or instruments, although the same reference standards for biochemical parameters were employed. Keeping this in mind, we adjusted our logistic regression analyses for the Centre of enrolment. Furthermore, a cohort of newly diagnosed CP patients without prior medical counselling regarding lifestyle or dietary interventions might reflect the investigated association better. However, given the relatively low incidence and prevalence of CP^45^, it is difficult to investigate a sufficient sample size of these patients. Also, the cross-sectional study design precludes the possibility to establish any cause-effect relationship, and we can only report an association between certain parameters and the presence of osteopathy, useful to generate hypotheses that need validation. As far as regards the method employed for vitamin K measure, while HPLC is currently considered the elective technique to measure vitamin K^46^, this remains a complex determination and the measure of markers of vitamin K activity such as undercarboxylated OC or protein induced by vitamin K absence (PIVKA) might have been helpful. However, as OC levels are also influenced by vitamin D and PTH levels^46^, which can be altered in CP patients, the concomitant measure of both vitamin K and these markers should be considered in future studies. Finally, as vitamin K is mainly transported by triglyceride-rich lipoproteins, the lack of triglyceride measure in the present study is a limitation as it does not allow normalization of vitamin K values.

In conclusion, findings of the present study confirm the high rate of osteopathy in a large cohort of patients with CP and reinforce the recommendation to screen CP patients with DXA, in order to prevent pathological fractures. The present findings also suggest, for the first time, that specifically in male patients, who typically represent the majority of CP subjects, vitamin K deficiency is the most relevant factor associated with osteoporosis. As this study can only demonstrate an association and not causality, further investigation on the role of vitamin K on bone turnover in the setting of CP are necessary. Future studies should also investigate the association between dietary patterns and vitamins intake and clinically relevant outcomes associated with nutritional status and vitamin levels, such as risk of hospitalization for fractures, infections and cardiovascular events, in CP patients.

## Study Highlights

### What is current knowledge

- In patients with chronic pancreatitis (CP) osteopathy has been reported to be common but the reported prevalence is heterogeneous and previous studies carry several limitations.
- Vitamin K, which plays a key role in bone metabolism, has never been investigated among factors associated with osteopathy in CP.

### What is new here

- This is the largest study aimed at investigating the prevalence of osteopathy in patients with chronic pancreatitis with standardized criteria to assess disease severity and pancreatic exocrine insufficiency (PEI).
- A high rate of osteopenia and osteoporosis was confirmed, alongside vitamin D deficiency and PEI, that were not associated with osteopathy.
- Female sex, age and BMI were associated with of osteoporosis in the whole cohort, while in male patients, the only factor associated with osteoporosis was vitamin K deficiency.

## Electronic supplementary material

Supplementary Information

Supplementary Table 2

Supplementary Table 2
