# Supplementary material for: Vitamins D and K as Factors Associated with Osteopathy in Chronic Pancreatitis: A Prospective Multicentre Study (P-BONE Study)
Source: Clin Transl Gastroenterol. 2018 Oct 15;9(10):197. doi: 10.1038/s41424-018-0066-8 (PMC6189068; doi:10.1038/s41424-018-0066-8)
Supplement: Supplementary file 1 — Supplementary Information [file 41424_2018_66_MOESM1_ESM.docx]

**Supplementary material**

*Vitamin K measurement*

500 μl of serum sample were added to 10 μl of the IS (Vitamin K-d7, 100 ng ml-1 in ethanol) and then diluted with 1,5 ml of cold Ethanol in order to denature the proteins. Samples were vortexed for one minute. 500 µl of isopropanol and 1,5 ml of hexane were added to the sample mixture and centrifuged at 14,000 × g for 5 min at 0°C. Later, the upper hexane clean layers were collected. The extraction process was repeated twice. The hexane fractions (3 mL) were dried in a speed vacuum system and resuspended in 70 µl of 0.1% aqueous formic acid with 30% of methanol and finally centrifuged for 10 min at 4000 rpm at 0°C. The supernatants were transferred in vials for the autosampler and 30 μl were injected into the HPLC system for analysis.

The chromatographic analysis was performed on a Shimadzu NEXERA X2 Ultra High Performance Liquid Chromatography (Shimadzu, Kyoto, Japan) system equipped with controller (CBM-20A), pump (LC-30AD), autosampler (SIL-30AC), online degasser (DGU-20A), and column heater (CTO-30A). The chromatographic separations were performed on a Kinetex® C18 LC Column 50 x 2.1 mm, 2.6 µm 100 Å (Phenomenex, Torrance, CA, USA) maintained at a temperature of 60°C. The mobile phases consisted of 0.1% aqueous formic acid with 30% methanol (A) and 100% methanol (B). Elution was performed in gradient conditions at flow rate of 0.6 mL/min using 40% eluent B for 7 min, 100% eluent B for 3 min, and afterwards re-equilibrated with eluent A for 7 min. Injection volume was 30 µL. All sample extracts were maintained in the autosampler at 4°C awaiting injection. A QTRAP® 5500 tandem mass spectrometer (AB Sciex, Concord, Ontario, Canada) with Atmospheric Pressure Chemical Ionisation (APCI) source was used in Multiple Reaction Monitoring mode. Data analysis were obtained and processed by Analyst 1.6.3 software. The MS/MS conditions for Vitamin K1 and IS are reported in **Supplementary Table 1**

**Supplementary Table 1.** Monitored Ion Transitions and Mass Spectrometry Parameters

| Analyte (Precursor Ion)  (m/z, amu) | Fragment  (m/z, amu) | DP  (V) | EP  (V) | CE  (V) | CXP  (V) |
| --- | --- | --- | --- | --- | --- |
| Vitamin K1 (451.40) | 187.1 | 65 | 12 | 32 | 15 |
|  | 128.0 | 65 | 12 | 103 | 9.0 |
|  | 199.0 | 65 | 12 | 39 | 5.0 |
| Vitamin K-d7 (458.4) | 194.3 | 65 | 12 | 35 | 18 |

DP: declustering potential; EP: entrance potential; CE: collision energy; CXP: collision cell exit potential.
