# Supplementary material for: Vitamins D and K as Factors Associated with Osteopathy in Chronic Pancreatitis: A Prospective Multicentre Study (P-BONE Study)
Source: Clin Transl Gastroenterol. 2018 Oct 15;9(10):197. doi: 10.1038/s41424-018-0066-8 (PMC6189068; doi:10.1038/s41424-018-0066-8)
Supplement: Supplementary file 2 — Supplementary Table 2 [file 41424_2018_66_MOESM2_ESM.docx]

**Supplementary Table 2.** Exclusive deficiency of either Vitamin D or Vitamin K and their interaction among 128 male chronic pancreatic patients with or without osteoporosis.

|  | **Osteoporosis**  **(n=18)** | **No Osteoporosis**  **(n=110)** | **Multivariable analysis*^1^***  **OR (95% CI)** | ***P* value** |
| --- | --- | --- | --- | --- |
| **Exclusive or combined Deficiency** |  |  |  |  |
| Neither Vit D or K deficiency | 6 (33.3%) | 37 (33.6%) | 1.00 | - |
| Vit D deficiency only | 4 (22.2%) | 46 (38.9%) | 0.46 (0.12-1.80) | 0.26 |
| Vit K deficiency only | 5 (27.7%) | 7 (5.9%) | 9.21 (1.89-44.79) | 0.005 |
| Interaction | 3 (16.6%) | 20 (18.1%) | 1.34 (0.27-6.58) | 0.71 |

OR: Odds Ratio, CI: Confidence Interval

*^1^*Odds Ratios adjusted for age, body mass index, PTH levels and Centre of enrollment.
