# Supplementary material for: Vitamins D and K as Factors Associated with Osteopathy in Chronic Pancreatitis: A Prospective Multicentre Study (P-BONE Study)
Source: Clin Transl Gastroenterol. 2018 Oct 15;9(10):197. doi: 10.1038/s41424-018-0066-8 (PMC6189068; doi:10.1038/s41424-018-0066-8)
Supplement: Supplementary file 3 — Supplementary Table 2 [file 41424_2018_66_MOESM3_ESM.docx]

**Supplementary Table 2.** Logistic regression analysis for factors associated with osteoporosis in the 69 female patients with CP.

| Univariate* | | | Multivariate* | |
| --- | --- | --- | --- | --- |
|  | OR (95% CI) | p value | OR (95% CI) | p value |
| Age (per increasing year) | 1.08 (1.02-1.13) | 0.002 | 1.09 (1.03-1.16) | 0.002 |
| BMI (per increasing unit) | 0.84 (0.73-0.96) | 0.01 | 0.86 (0.73-1.01) | 0.08 |
| PTH (per increasing increasing unit) | 1.01 (0.99-1.03) | 0.11 | - |  |
| Vitamin D deficiency | 0.66 (0.17-2.50) | 0.54 | - |  |
| Vitamin K deficiency | 0.16 (0.01-1.38) | 0.1 | - |  |
| CRP (per increasing unit) | 1.03 (0.90-1.18) | 0.61 | - |  |
| Faecal elastase (per increasing unit) | 0.99 (0.99-1.00) | 0.43 | - |  |
| Diabetes | 1.24 (0.45-3.42) | 0.67 | - |  |
| Active Smoking | 2.51 (0.84-7.46) | 0.09 | - |  |
| Advanced-marked disease | 0.70 (0.12-3.95) | 0.68 | - |  |
| Alcholic Etiology | 3.69 (0.95-14.25) | 0.06 | 2.54 (0.55-11.72) | 0.23 |
| Disease duration (per year) | 1.00 (0.99-1.00) | 0.53 | - |  |
| PERT | 1.18 (0.43-3.23) | 0.73 | - |  |

*Adjusted for centre of enrolment. BMI: body mass index; PTH: parathormone; CRP: C-reactive protein; PERT: Pancreatic Enzyme Replacement Therapy
